# Anti-Amnesic Effect of Synbiotic Supplementation Containing *Corni fructus* and *Limosilactobacillus reuteri* in DSS-Induced Colitis Mice

**DOI:** 10.3390/ijms24010090

**Published:** 2022-12-21

**Authors:** Hyo Lim Lee, Jong Min Kim, Jong Hyun Moon, Min Ji Kim, Hye Rin Jeong, Min Ji Go, Hyun-Jin Kim, Hyun Ji Eo, Uk Lee, Ho Jin Heo

**Affiliations:** 1 Division of Applied Life Science (BK21), Institute of Agriculture and Life Science, Gyeongsang National University, Jinju 52828, Republic of Korea; 2 Division of Special Forest Resources, Department of Forest Bioresources, National Institute of Forest Science (NIFoS), Suwon 16631, Republic of Korea

**Keywords:** *Corni fuctus*, *Limosilactobacillus reuteri*, cognitive impairment, gut microbiota, anti-inflammation, gut-brain axis

## Abstract

This study was conducted to compare the synbiotic activity between *Corni fructus* (*C. fructus*) and *Limosilactobacillus reuteri* (*L. reuteri*) on dextran sulfate sodium (DSS)-induced colitis and cognitive dysfunction in C57BL/6 mice. *C. fructus* (as prebiotics, PRE), *L. reuteri* (as probiotics, PRO), and synbiotics (as a mixture of *L. reuteri* and *C. fructus*, SYN) were fed to mice for 3 weeks. Consumption of PRE, PRO, and SYN ameliorated colitis symptoms in body weight, large intestinal length, and serum albumin level. Moreover, SYN showed a synergistic effect on intestinal permeability and intestinal anti-inflammation response. Also, SYN significantly improved cognitive function as a result of measuring the Y-maze and passive avoidance tests in DSS-induced behavioral disorder mice. Especially, SYN also restored memory function by increasing the cholinergic system and reducing tau and amyloid β pathology. In addition, PRE, PRO, and SYN ameliorated dysbiosis by regulating the gut microbiota and the concentration of short-chain fatty acids (SCFAs) in feces. The bioactive compounds of *C. fructus* were identified with quinic acid, morroniside, loganin, and cornuside, using ultra-performance liquid chromatography-quadrupole time-of-flight tandem mass spectrometry (UPLC-Q-TOF-MS^2^). In conclusion, synbiotic supplementation alleviated DSS-induced colitis and cognitive dysfunction by modulating gut microbiota, proinflammatory cytokines, and SCFAs production.

## 1. Introduction

Inflammatory bowel disease (IBD), such as ulcerative colitis (UC) and Crohn’s disease (CD), is a chronic intestinal inflammation characterized by weight loss, fecal bleeding, abdominal pain, and loose stool [1]. Many studies have reported that it is closely connected with gut microbiome dysbiosis, mucosal immune response, intestinal barrier disruption, and genetic factors [2,3]. Intestinal barrier damage increases intestinal permeability and disrupts the intestinal mucosal immune system, leading to the overproduction of oxidative stress and up-regulation of inflammatory cytokines [4]. Moreover, gut microbiome dysbiosis causes an increase of bacterial toxin lipopolysaccharide (LPS) and releases inflammatory cytokines through the toll-like receptor 4 (TLR4)/nuclear factor-κB (NF-kB) signaling pathway [5]. Inflammatory cytokines from the gut can reach the brain via systemic circulation, and lead to neuroinflammation and neuronal apoptosis by increasing neurotoxic substances, such as nitric oxide and oxygen radicals [6]. Thus, gut microbiome dysbiosis might contribute to the pathogenesis of neurodegenerative disease, such as Alzheimer’s disease (AD), through inflammation derived from intestinal inflammatory pathways [7]. Indeed, many studies have found an increased incidence of psychiatric and cognitive impairments with chronic inflammation in IBD patients [8]. In addition, growing evidence has been reported that the gut modulates host immunity, metabolism, and behavior through direct and indirect chemical signals with the nervous system [8,9]. To suppress the onset and progress of IBD contributing to cognitive impairment, prebiotics and probiotics have recently been considered as an alternative, preventive, and corrective treatment strategy instead of pharmacological therapy with its potential side effects [10].

*Corni fructus* (*C. fructus*) has been used as herbal medicine in East Asia for more than 2000 years. It has been reported that *C. fructus* has various bioactive activities, such as anti-microbial, neuroprotective, anti-allergic, and anti-diabetic effects [11,12,13,14]. The main components of *C. fructus* include iridoid glycosides, secoiridoid glycoside compounds, bisiridoid glycosides, triterpenoid, and tannin-based compounds [15,16]. Especially the iridoid glycosides, such as morroniside, loganin, and 7-O-galloyl-D-sedoheptulose, also have been reported to have a protective effect on colitic mice [16].

*Limosilactobacillus reuteri* (*L. reuteri*) is a well-studied probiotic bacterium found in a variety of organs, including the gastrointestinal tract [17], and considered to be one of a few native *Limosilactobacillus* species in the human intestine. It is reported that *L. reuteri* not only produces anti-bacterial molecules, including reuterin, but also maintains intestinal microbial homeostasis due to its ability to improve intestinal barrier function and modulate the host’s immune response [18,19]. *L. reuteri* can ferment carbohydrates to produce short-chain fatty acids (SCFAs) that are beneficial for enhancing immune regulation and colonic epithelial permeability [9]. In addition, SCFAs are involved in microglia maturation by providing energy to cells, as well as regulating the function of the blood-brain barrier (BBB) [20]. It may also affect the brain by affecting the expression of cognition-related signaling molecules, such as serotonin transporter, brain-derived neurotrophic factor (BDNF), and gut hormone peptide YY (PYY) [21]. Although various physiological activities of *C. fructus* and *L. reuteri* have been reported, synergistic effects on anti-amnesic effect and intestinal health, including an imbalance of gut microbiota, are insufficient. Our previous in vitro study confirmed that *C. fructus* promoted the growth of *L. reuteri,* widely known as probiotics related to the gut-brain axis [22]. Therefore, this study was conducted to evaluate whether *C. fructus* has prebiotic activity and synergistic effects with *L. reuteri* on cognitive impairment caused by dextran sulfate sodium (DSS)-induced colitis in C57BL/6 mice.

## 2. Results

### 2.1. Effect of PRE, PRO, and SYN on Pathological Symptoms of Colitis

**Figure 1:**
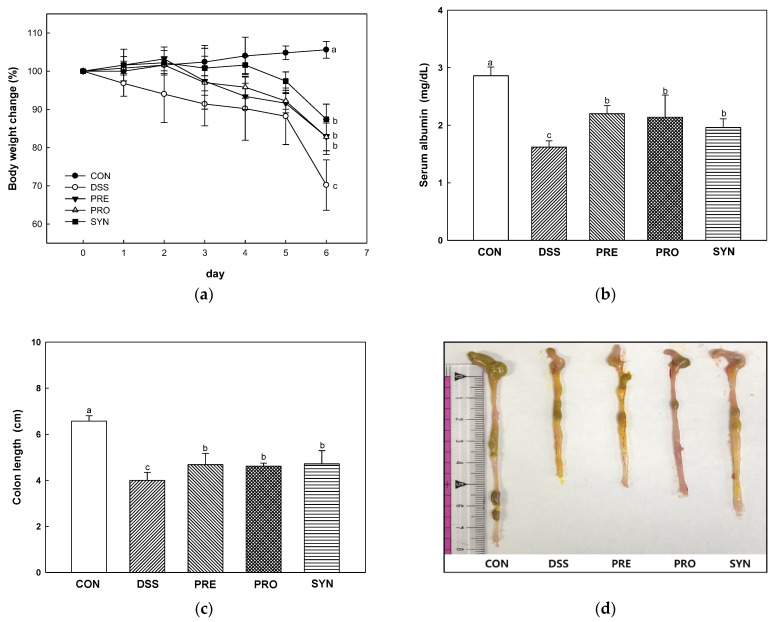
Effect of PRE, PRO, and SYN on pathological symptoms of DSS-induced colitis mice. The body weights change (**a**), serum albumin content (**b**), colon length (**c**), and macroscopic appearance (**d**). The results were presented as the mean ± SD (*n* = 5). Data were statistically considered at *p* < 0.05, and different lowercase letters indicate significant differences between groups.

To determine the effects of PRE, PRO, and SYN on the symptoms of colitis, body weight change, colon length, and serum albumin content were measured (Figure 1). The body weight change rate was decreased in the DSS group (70.00%) compared with the CON group (105.06%) (Figure 1a). However, the body weight change of the PRE, PRO, and SYN groups (82.80%, 82.80%, and 87.40%, respectively) was increased compared to that of the DSS group (Figure 1a). Serum albumin content was decreased in the DSS group (1.62 mg/dL) compared to the CON group (2.86 mg/dL) (Figure 1b). However, the serum albumin content of the PRE, PRO, and SYN groups (2.2 mg/dL, 2.14 mg/dL, and 1.96 mg/dL, respectively) was increased compared to that of the DSS group. In addition, colon length was decreased in the DSS group (4.00 cm) compared with the CON group (6.57 cm) (Figure 1c,d). However, the colon length of the PRE, PRO, and SYN groups (4.68 cm, 4.62 cm, and 4.72 cm, respectively) was increased compared to that of the DSS group.

### 2.2. Effect of PRE, PRO and, SYN on Intestinal Permeability

**Figure 2:**
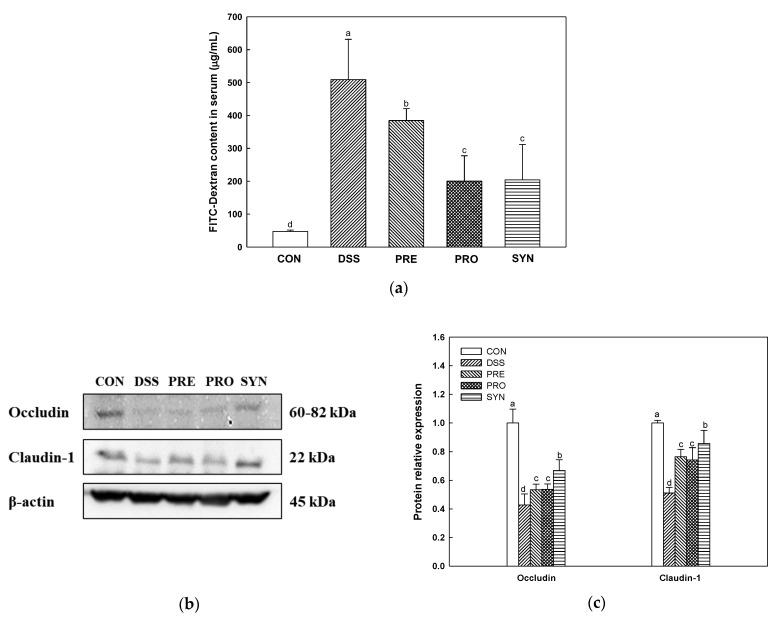
Effects of PRE, PRO, and SYN on permeability and junction proteins of DSS-induced colitis mice. Fluorescein isothiocyanate (FITC)-dextran levels in serum (**a**), Western blot band images (**b**), and the expression levels of tight junction proteins; occludin, and claudin-1 (**c**). The results were presented as the mean ± SD (FITC-dextran; *n* = 5, Western blot; *n* = 3). Data were statistically considered at *p* < 0.05, and different lowercase letters indicate significant differences between groups.

Intestinal permeability was confirmed by measuring fluorescein-5-isothiocyanate (FITC)-dextran content in serum and the expression levels of tight junction (TJ) protein in the colon tissue (Figure 2). FITC-dextran content was increased in the DSS group (509.19 μg/mL) compared to the CON group (47.24 μg/mL) (Figure 2a). However, the FITC-dextran content of the PRE, PRO, and SYN groups (384.48 μg/mL, 200.28 μg/mL, and 204.50 μg/mL, respectively) was reduced compared to that of the DSS group.

The results of TJ protein in the colon tissue are shown in Figure 2b,c. The expression levels of occludin (0.43) and claudin-1 (0.51) of the DSS group were decreased compared with the CON group (1.00). However, the expression levels of occludin (0.53, 0.54, and 0.67, respectively) and claudin-1 (0.76, 0.74, and 0.86, respectively) of the PRE, PRO, and SYN groups were increased compared to that of the DSS group.

### 2.3. Effect of PRE, PRO, and SYN on Inflammatory Responses in Colon Tissue

**Figure 3:**
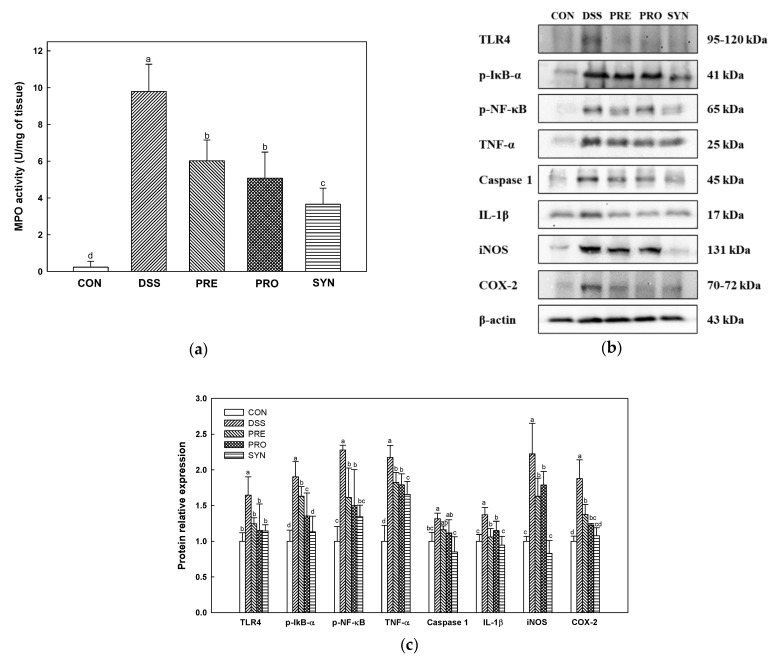
Effects of PRE, PRO, and SYN on inflammatory responses in colon tissues of DSS-induced colitis mice. Myeloperoxidase (MPO) activity (**a**), Western blot band images (**b**), and the expression levels of TLR4, p-IκB-α, p-NF-κB, TNF-α, caspase 1, IL-1β, iNOS, and COX-2 (**c**). The results were presented as the mean ± SD (MPO activity; *n* = 5, Western blot; *n* = 3). Data were statistically considered at *p* < 0.05, and different lowercase letters indicate significant differences between groups.

Myeloperoxidase (MPO) activity was increased in the DSS group (9.79 U/mg) compared with the CON group (0.24 U/mg) (Figure 3a). However, the MPO activity of the PRE, PRO, and SYN groups (6.01 U/mg, 5.07 U/mg, and 3.66 U/mg, respectively) was down-regulated compared to that of the DSS group.

The measurement results of inflammatory protein expression are shown in Figure 3b,c. TLR4, phospho- nuclear factor of kappa light polypeptide gene enhancer in B-cells inhibitor-alpha (p-IκBα), p-NF-κB, tumor necrosis factor-α (TNF-α), caspase 1, interleukin-1β (IL-1β), inducible nitric oxide synthase (iNOS), and cyclooxygenase-2 (COX-2) were increased in the DSS group (1.65, 1.90, 2.28, 2.18, 1.31, 1.37, 2.22, and 1.88, respectively) compared with the CON group (1.00). However, the expression of these inflammatory proteins was decreased in the PRE, PRO, and SYN groups (TLR4; 1.25, 1.15, and 1.14, p-IκBα; 1.63, 1.35, and 1.13, p-NF-κB; 1.61, 1.50, and 1.34, TNF-α; 1.82, 1.79, and 1.66, Caspase 1; 1.16, 1.11 and, 0.85, IL-1β; 1.14, 1.15, and 0.94, iNOS; 1.63, 1.79, and 0.83, COX-2; 1.37, 1.23, and 1.08, respectively) compared to that of the DSS group.

### 2.4. Effect of PRE, PRO, and SYN on Gut Microbiota Composition

**Figure 4:**
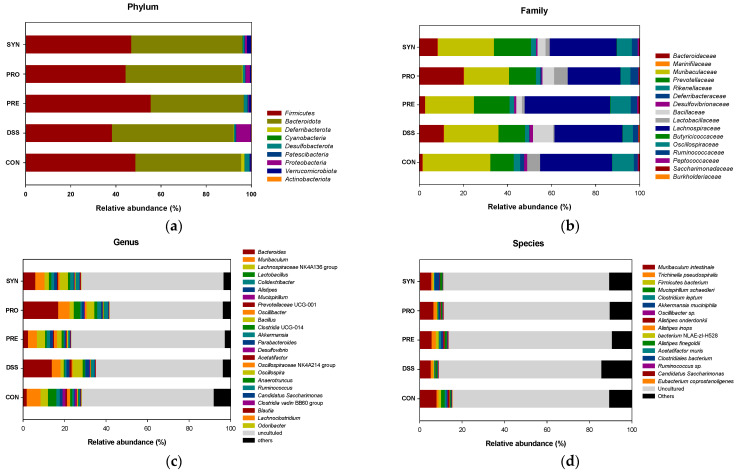
Effects of PRE, PRO, and SYN on gut microbiome composition of DSS-induced colitis mice. Relative abundance (%) at the phylum (**a**), family (**b**), genus (**c**), and species (**d**) level of each group. The results were presented as the mean ± SD (*n* = 3). Data were statistically considered at *p* < 0.05, and different lowercase letters indicate significant differences between groups.

To account for the effects of the PRE, PRO, and SYN treatments on the mice microbiome, relative abundances were measured (Figure 4 and Table 1). The taxonomic breadth and abundance of bacteria identified in the mice feces were visualized through Krona plots (Figure 4a and Supplementary Figure S1). The bar charts in Figure 4a–d show the overall changing pattern of the top 10% most abundant bacteria in the feces.

As shown in Figure 4b, the most abundant phyla were confirmed as *Firmicutes* and *Bacteroidota*. At the phylum level, the relative abundance of *Proteobacteria* was significantly increased in the DSS group (9.19%) compared to the CON group (0.08%). However, the relative abundance of *Proteobacteria* of the PRE, PRO, and SYN groups (0.31%, 1.75%, and 0.63%, respectively) were significantly reduced compared to that of the DSS group. In addition, the *Firmicutes*/*Bacteroidota* ratio was significantly decreased in the DSS group (0.61%) compared to the CON group (1.39%). However, the ratio was significantly restored in the PRE, PRO, and SYN groups (1.36%, 0.90%, and 1.04%, respectively).

At the family level, the relative abundance of *Bacillacea* was significantly increased in the DSS group (6.93%) compared to the CON group (0.06%) (Figure 4c). The relative abundance of *Lactobacillaceae* was significantly reduced in the DSS group (0.46%) compared to the CON group (5.56%). However, the relative abundance of *Lactobacillaceae* of the PRO group (6.05%) was increased compared to that of the DSS group.

At the genus level, 8 genera were detected, and the relative abundance of *Bacillus* and *Prevotellaceae* UCG-001 were significantly increased in the DSS group (6.14% and 2.31%) compared to the CON group (0.06% and 1.01%). However, the relative abundance of *Prevotellaceae* UCG-001 of the PRE, PRO, and SYN groups (0.81%, N.D., and 0.65%, respectively) was significantly decreased compared to that of the DSS group. Whereas, *Odoribacter*, *Lactobacillus*, *Lactococcus*, *Lachnospiraceae* NK4A136 group, *Anaerotruncus*, and *Akkermasia* were significantly decreased in the DSS group (0.04%, 0.70%, 0.23%, 1.43%, 0.06%, and 0.21%, respectively) compared to the CON group (0.31%, 8.89%, 0.53, % 2.90%, 0.66%, and 0.43%, respectively). However, the PRE, PRO, and SYN treatment were regulated these bacteria as *Lactococcus* (0.98%, 0.65%, and 0.87%, respectively), *Lachnospiraceae* NK4A136 group (3.59%, 0.41%, and 1.68%, respectively), *Anaerotruncus* (0.14, 0.81, and 0.19, respectively), and *Akkermasia* (0.97%, 0.49%, and 2.22%, respectively) compared to that of the DSS group. The relative abundance of *Lactobacillus* was significantly increased in the PRO group (7.41%) compared to the DSS group (0.70%).

At the species level, 2 species were identified, and the relative abundance of *Akkermensia mucinifila* and *Alistipes onderdonkii* were down-regulated in the DSS group (0.30% and 0.02%) compared with the CON group (0.45 and 0.33). However, the relative abundance of *Akkermensia mucinifila* and *Alistipes onderdonkii* of the PRE, PRO, and SYN groups (*Akkermensia mucinifila*, 0.85%, 0.55%, and 3.35%; *Alistipes onderdonkii*, 0.34%, 0.19%, and 0.28%, respectively) were significantly up-regulated compared to that of the DSS group.

### 2.5. Effect of PRE, PRO, and SYN on Concentration of Short-Chain Fatty Acids (SCFAs) in Fecal

The results of fecal SCFAs concentration are presented in Table 2. Acetic acid content was decreased in the DSS group (81.76 mM/g) compared with the CON group (182.93 mM/g). However, the acetic acid content of the PRE, PRO, and SYN groups (216.39 mM/g, 200.44 mM/g, and 254.22 mM/g, respectively) was increased compared to that of the DSS group. Propionic acid content was down-regulated in the DSS group (8.85 mM/g) compared with the CON group (18.44 mM/g). However, the propionic acid content of the PRE, PRO, and SYN groups (28.04 mM/g, 25.79 mM/g, and 30.12 mM/g, respectively) were up-regulated in the DSS group. Butyric acid content was reduced in the DSS group (7.28 mM/g) compared with the CON group (39.47 mM/g). However, the butyric acid content of the PRE, PRO, and SYN groups (23.20 mM/g, 24.83 mM/g, and 38.18 mM/g, respectively) were elevated compared to that of the DSS group. Peculiarly, the SCFAs concentration in the SYN group was significantly larger in comparison to the PRE and PRO groups.

### 2.6. Effect of PRE, PRO, and SYN on Behavioral Disorder

**Figure 5:**
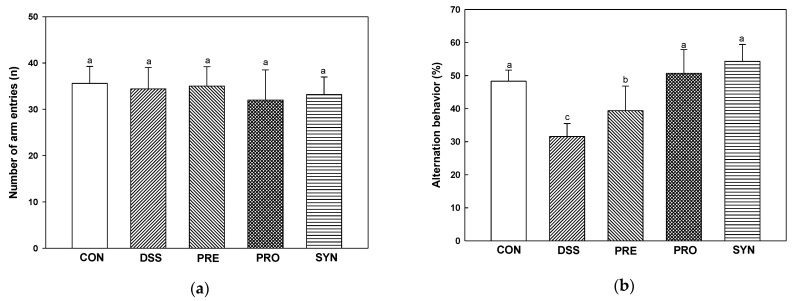

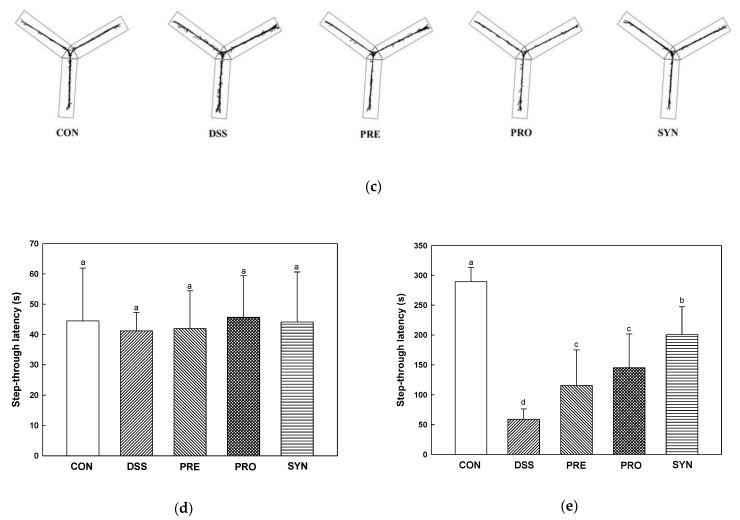
Effects of PRE, PRO, and SYN on learning and memory impairment of DSS-induced colitis mice. Number of arm entries (**a**), alternation behavior (**b**), and path motion (**c**) on Y-maze test, and latency during habituation (**d**) and step-through latency (**e**) on passive avoidance test. The results were presented as the mean ± SD (*n* = 5). Data were statistically considered at *p* < 0.05, and different lowercase letters indicate significant differences between groups.

Using the Y-maze test (Figure 5a–c), the number of arm entries in all groups showed no significant difference in the motor abilities of each mouse (Figure 5a). Alternation behavior was decreased in the DSS group (31.55%) compared with the CON group (48.30%). However, the alternation behavior of the PRE, PRO, and SYN groups (39.40%, 50.71%, and 54.32%, respectively) was increased compared to that of the DSS group (Figure 5a–c).

Short-term memory was evaluated using the passive avoidance test (Figure 5d,e). On the first day, there was no significant difference in the time to enter the dark chamber from the light chamber in all groups (Figure 5d). On the second day, step-through latency was reduced in the DSS group (58.90 s) compared to the CON group (289.40 s). In contrast, the step-through latency of the PRE, PRO, and SYN groups (115.60 s, 144.95 s, and 200.62 s, respectively) were increased compared to that of the DSS group (Figure 5e).

### 2.7. Effect of PRE, PRO, and SYN on Antioxidant Parameters in Brain Tissue

**Figure 6:**
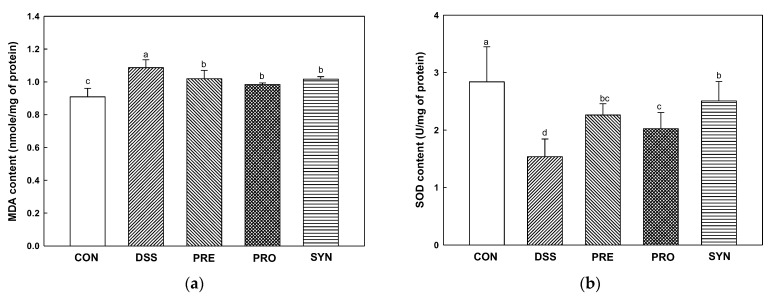
Effects of PRE, PRO, and SYN on antioxidant system of DSS-induced colitis mice. Malondialdehyde (MDA) content (**a**) and superoxide dismutase (SOD) level (**b**) in brain tissues. The results were presented as the mean ± SD (*n* = 5). Data were statistically considered at *p* < 0.05, and different lowercase letters indicate significant differences between groups.

The Malondialdehyde (MDA) content was increased in the DSS group (1.09 nmole/mg of protein) compared with the control group (0.91 nmole/mg of protein) (Figure 6a). However, the MDA content of the PRE, PRO, and SYN groups (1.01 nmole/mg of protein, 0.98 nmole/mg of protein, and 1.02 nmole/mg of protein, respectively) was decreased compared to that of the DSS group.

The Superoxide dismutase (SOD) level was reduced in the DSS group (1.54 U/mg of protein) compared to the CON group (2.84 U/mg of protein) (Figure 6b). However, the SOD level of the PRE, PRO, and SYN groups (2.26 U/mg protein, 2.02 U/mg protein and 2.51 U/mg protein, respectively) was increased compared to that of the DSS group.

### 2.8. Effect of PRE, PRO, and SYN on Cholinergic System

**Figure 7:**
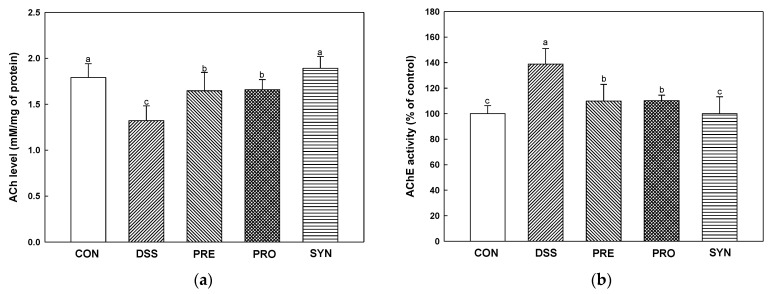

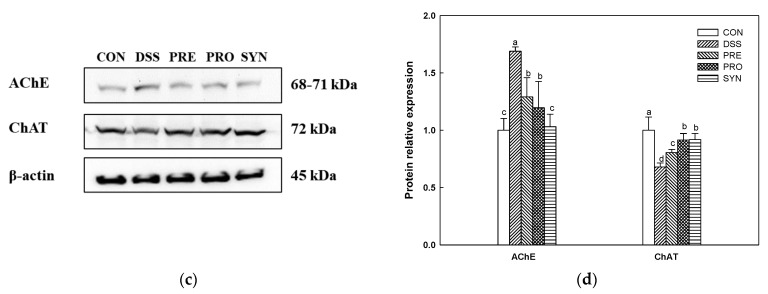
Effects of PRE, PRO, and SYN on cognitive impairment of DSS-induced colitis mice via regulation of cholinergic system. Acetylcholine (ACh) content (**a**), acetylcholinesterase (AChE) activity (**b**), Western blot band images (**c**), and the expression levels of AChE and choline acetyltransferase (ChAT) (**d**). The results were presented as the mean ± SD (ACh content and AChE activity, *n* = 5; Western blot, *n* = 3). Data were statistically considered at *p* < 0.05, and different lowercase letters indicate significant differences between groups.

Acetylcholine (ACh) content was decreased in the DSS group (1.32 mM/mg of protein) compared with the CON group (1.79 mM/mg of protein) (Figure 7a). However, the ACh content of the PRE, PRO, and SYN groups (1.65 mM/mg of protein, 1.66 mM/mg of protein, and 1.89 mM/mg of protein, respectively) was increased compared to that of the DSS group. Acetylcholinesterase (AChE) activity was increased in the DSS group (138.88%) compared to the CON group (100.00%) (Figure 7b). However, the AChE activity of the PRE, PRO, and SYN groups (109.94%, 110.20%, and 99.94%, respectively) was decreased compared to that of the DSS group. Similarly, the expression of AChE was also increased in the DSS group (1.68) compared with the CON group (1.00) (Figure 7c,d). However, the expression of AChE of the PRE, PRO, and SYN groups (1.29, 1.20, and 1.03, respectively) was decreased compared to that of the DSS group. In contrast, the expression of choline acetyltransferase (ChAT) was reduced in the DSS group (0.68) compared with the CON group (1.00). However, the expression of ChAT of the PRE, PRO, and SYN groups (0.81, 0.91, and 0.92, respectively) was increased compared to that of the DSS group.

### 2.9. Effect of PRE, PRO, and SYN on Phosphorylated-Tau (p-Tau) and Amyloid β (Aβ) Protein Expression

**Figure 8:**
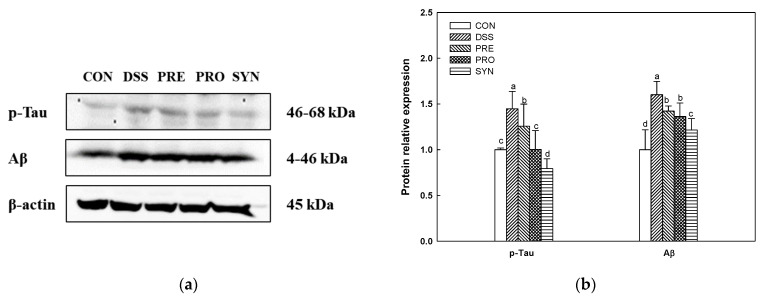
Effects of PRE, PRO, and SYN on cognitive impairment of DSS-induced colitis mice via inhibition of tau hyperphosphorylation and amyloid β (Aβ) production. Western blot band images (**a**), and the expression levels of p-tau and Aβ (**b**). The results were presented as the mean ± SD (*n* = 3). Data were statistically considered at *p* < 0.05, and different lowercase letters indicate significant differences between groups.

The expression of p-tau (1.45) and Aβ (1.60) was increased in the DSS group compared with the CON group (1.00) (Figure 8). However, the expression of p-tau (1.26, 1.00, and 0.79, respectively) and Aβ (1.42, 1.24, and 1.22, respectively) of the PRE, PRO, and SYN groups was decreased compared to that of the DSS group.

### 2.10. Effect of PRE, PRO, and SYN on Apoptosis Pathway in Brain Tissue

**Figure 9:**
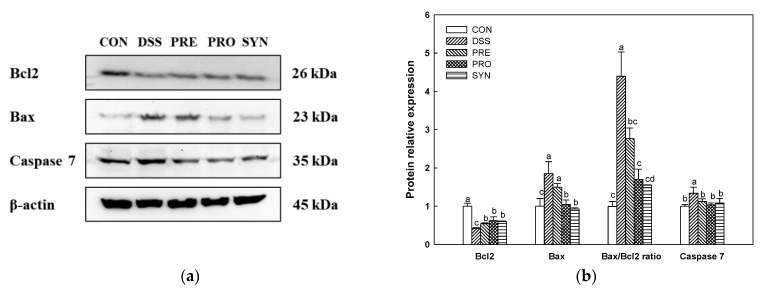
Effects of PRE, PRO, and SYN on cognitive impairment of DSS-induced colitis mice via regulation of apoptosis pathway. Western blot band images (**a**), and the expression levels of Bcl2, Bax, Bax/Bcl2 ratio, and caspase 7 (**b**). The results were presented as the mean ± SD (*n* = 3). Data were statistically considered at *p* < 0.05, and different lowercase letters indicate significant differences between groups.

The expression of B-cell lymphoma-2 (Bcl2) was decreased in the DSS group (0.42) compared to the CON group (1.00) (Figure 9). However, the expression of Bcl2 of the PRE, PRO, and SYN groups (0.54, 0.62, and 0.60, respectively) was increased compared to that of the DSS group. The expression of Bcl2-associated X protein (Bax) in the DSS group (1.85) was increased compared with the CON group (1.00). However, the expression of Bax of the PRE, PRO, and SYN groups (1.50, 1.05, and 0.93, respectively) was decreased compared to that of the DSS group. In addition, the Bax/Bcl2 ratio also increased in the DSS group (4.39) compared to the CON group (0.99). However, the Bax/Bcl2 ratio of the PRE, PRO, and SYN groups (2.77, 1.70, and 1.55, respectively) was reduced compared to that of the DSS group. The expression of caspase-7 in the DSS group (1.34) was increased compared with the CON group (1.00). However, the expression of caspase-7 of the PRE, PRO, and SYN groups (1.12, 1.04, and 0.92, respectively) was decreased compared to that of the DSS group.

### 2.11. Bioactive Compounds of Water Extract of C. fructus (WCF)

**Figure 10:**
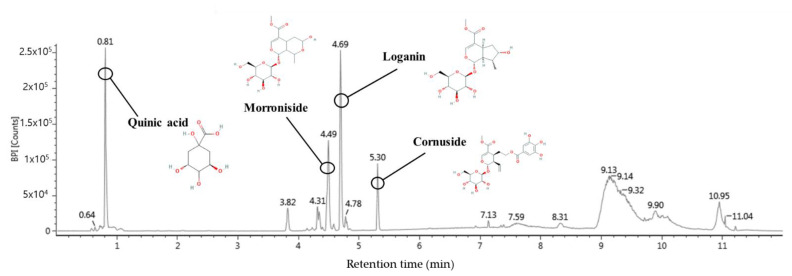
UPLC-Q-TOF/MS chromatogram of the ethyl acetate fraction from *Corni fructus* (WCF).

Ultra-performance liquid chromatography-quadrupole time-of-flight tandem mass spectrometry (UPLC-Q-TOF/MS^2^) in negative ion mode detected identifiable chromatographic peaks (Figure 10), including four bioactive compounds in WCF. The main detected compounds were identified by comparing the MS^2^ main fragments from previous literature reports and the Massbank database (Figure 10 and Table 3). A total of four compounds were tentatively identified by MS fragmentations. Quinic acid (retention time, 0.81 min; MS^2^ fragment, 85, 93, 109, 127, and 191 *m*/*z*), morroniside (retention time, 4.49 min; MS^2^ fragment, 101, 123, 141, 155, 243, and 451 *m*/*z*), loganin (retention time, 4.69 min; MS^2^ fragment, 101, 127, 209, 227, and 435 *m*/*z*), and cornuside (retention time, 5.30 min; MS^2^ fragment, 125, 169, 347, and 541 *m*/*z*) were identified as the main compounds (Table 3).

## 3. Discussion

Recently, as interest in gut health grows, research on the interaction between the gut and brain is also being actively conducted [23]. The gut and brain are complex and closely connected, and these interactions affect not only the gastrointestinal function, but also play important roles in the emotional and cognitive functions of the brain [24]. Gut dysbiosis, as one of the characteristics of IBD, can systemically contribute to the development of various diseases, such as cognitive disorders, through the gut-brain axis [25]. Prebiotics and probiotics have been used as strategies to recover from these various diseases [26]. However, recently synbiotics, which has the characteristics of prebiotics and probiotics, is considered a promising strategy for the treatment of IBD due to its synergistic effect related to the improvement of the gut microbiota imbalance and the inflammation response [27]. Therefore, this study was conducted to evaluate the synbiotic effect of PRE and PRO against DSS-induced colitis and cognitive decline.

Damage to intestinal epithelial cells (IECs) caused by colitis reduces the intestinal barrier function and induces various inflammatory reactions [28]. The IECs are easily destroyed during intestinal inflammation in IBD patients [29,30]. Intestinal inflammation disturbs IECs linked as TJ proteins and interferes with maintaining intestinal homeostasis by regulating the penetration of bacteria and toxins through chemical and physical defense systems [30]. Increased intestinal permeability leads to loss of albumin in the feces, leading to a decrease in blood albumin, which is observed in cases of poor nutritional status [31]. It also associated with body weight loss and colon shortening in DSS-induced colitis mice [32]. In this study, the PRE, PRO, and SYN groups improved body weight change, serum albumin content, and colon length in colitis mice (Figure 1). Moreover, the PRE, PRO, and SYN groups improved DSS-induced intestinal permeability by increasing TJ protein expression in this study. In particular, SYN supplementation significantly improved the expression of occludin and claudin-1, indicating a protective effect on the intestinal barrier (Figure 2). Similar to this study, Ahl et al. [33] reported that *L. reuteri* enhanced intestinal epithelial barriers by increasing the expression of occludin and ZO-1 and increasing the thickness of the mucus layer in DSS-induced colitis mice. Moreover, *L. reuteri* LR1 protected the increase in permeability of the intestinal epithelial cell line 1 (IPEC-1) monolayer by *Escherichia coli* K88 and reduced the adhesion and invasion of coliforms [34]. In addition, the administration of loganin and morroniside as the physiological components of *C. fructus* (Figure 10) prevented epithelial barrier injury by increasing the expression of Muc2, ZO-1, claudin-3, occludin, and E-cadherin in DSS-induced colitis [35]. Therefore, it can be considered that PRE and PRO and their combination are prophylactic materials that protect the intestinal barrier function in colitis. This suggests that the beneficial effects of the SYN group in reducing disease severity by improving gut barrier function could be linked with the synergistic activities between the probiotic and prebiotic ingredients.

When various harmful bacteria and toxins penetrate the intestinal lumen due to the dysfunction of the intestinal barrier, inflammatory mediators increase in immune cells and the secretion of cytokines is promoted [6]. In addition, the continuous activation of such inflammation causes deadly damage to the intestine. If the inflammatory response expressed by harmful stimuli is excessively expressed and continues chronically, it will cause abnormalities in the body [36]. In IBD, it is known that an imbalance of T cells and inflammatory mediators continuously activate the inflammatory response to cause chronic tissue damage [37]. Harmful bacteria and toxins accelerate the inflammatory response by passing through the damaged IECs. LPS, a component of the outer membrane of Gram-negative bacteria, is recognized as a toxin that can indicate microbial infection. It is perceived by TLR4 and activates NF-κB, a signaling substance involved in inflammatory response, apoptosis, cell proliferation, and epithelial differentiation [29]. In addition, nitric oxide (NO) produced by iNOS in response to inflammatory cytokines, free radicals, and LPS increases the expression of COX-2 and accelerates the inflammatory response [36]. Disruption of the gut immune system interferes with balancing the host response to pathogens that are increased due to dysbiosis. In addition, excessive intestinal inflammation can stimulate the central nervous system (CNS) via the vagus nerve, and, in particular, the circulation of pro-inflammatory cytokines and activated immune cells can access the brain where the blood-brain barrier is damaged [38]. SYN was the most effective in demonstrating anti-inflammatory effects by reducing the expression of pro-inflammatory mediators, such as p-IkB-α, TNF-α, caspase 1, IL-1β, and iNOS in colon tissue (Figure 3). *L. reuteri* is a well-known probiotic strain with anti-inflammatory effects and has been reported to protect against DSS-induced intestinal inflammation [19,39]. It has been reported that the anti-inflammatory effect of *L. reuteri* is due to the inhibition of mRNA expression of TNF-α, COX-2, and IL-6 [40]. Moreover, *C. fructus* extract exhibited anti-inflammatory effects by inhibiting iNOS and COX-2 expression through reduced NF-κB activity in RAW254.7 cells [41]. Furthermore, it has been reported that cornuside not only down-regulates p38, ERK1/2, and JNK1/2 phosphorylation in RAW 264.7 cells (macrophage), but also reduces NF-κB activation [42]. Therefore, in this study, PRE and PRO are thought to reduce various inflammatory responses by inhibiting NF-κB activation caused by stimulation of TLR4.

Imbalance of the gut microbiome impedes the regulation of intestinal homeostasis and plays a role in exacerbating colitis [25]. Dysbiosis of gut microbiota plays an essential role in exacerbating the severity of UC, and gut microbiota modulation can ameliorate DSS-induced colitis [43,44]. In addition, the gut microbiome of animals and humans affect cognitive function through various communication mechanisms [24]. In particular, changes in neurotransmitters or neuromodulators, such as γ-aminobutyric acid (GABA), serotonin, and SCFAs produced by gut bacteria, can affect cognitive function [8]. In addition, an increase in harmful bacteria can affect neuronal cells through an enhance in the release of inflammatory substances in the blood [6]. Previous studies have reported that UC induces dysbiosis by increasing *Bacteroidetes* with decreasing *Firmicutes* in mice fecal samples [25,42]. Also, DSS treatment has been reported to interrupt the growth of probiotic, such as *Anaerotruncus*, *Lactobacillus*, and *Bifidobacterium* at the genus level [40]. This study also showed results consistent with that, and *Lactobacillus* was decreased in the feces of the DSS group (Figure 9 and Table 1). Moreover, *Bacillus* and *Prevotellaceae* UCG-001, which are found in the characterization of the gut microbiota in diseases related to neuropsychiatric disorders [45], were increased in the DSS group. PRE, PRO, and SYN restored levels of *Alisipes onderdonkii* and *Akkermasia muciniphila*, which are known to play important roles in several neuropsychiatric disorders, such as mental health, AD, and cognitive impairment, as well as protecting the intestinal mucus layer [21,46,47]. These results demonstrated that the cognitive impairment due to colitis is associated with the imbalance of the gut microbiota. It is thought to have influenced cognitive function and behavioral disorders through the gut microbiota-gut-brain axis. The gut microbiome and the brain interact through the systemic immune system due to circulating cytokines [6]. Based on this, it is presumed that PRE, PRO, and SYN restore dysbiosis due to intestinal barrier destruction and intestinal inflammation, which leads to the improvement of cognitive function.

SCFAs are fatty acids with six or less carbon atoms, and are major metabolites produced by gut microbes from polysaccharides, such as glycogen, resistant starch, and dietary fiber [48]. SCFAs contribute to suppressing the inflammatory response by regulating the production of inflammatory mediators [49]. In particular, butyric acid is a fuel used for colonic epithelial cell growth, and it increases the TJ protein to increase the firmness of the intestinal barrier [50]. In addition to their function in the gut, SCFAs can enter the brain via BBB and can affect the brain, either directly or indirectly through hormonal and immune pathways and neural pathways [21]. SCFAs also interact with the brain through immune and neural pathways to improve BBB integrity, affect neurotrophic factor levels, and directly activate vagus afferents to be involved in gut-brain communication [22]. In this study, the PRE, PRO, and SYN groups grew the abundance of *Akkermansia muciniphila*, *Odoribacter*, and *Lachnospiraceae* NK4A136 group known as SCFAs-producing bacteria [51,52,53]. These results may provide evidence for the increased SCFAs content in the PRE, PRO, and SYN groups, and it is considered that WCF and *L. reuteri* contribute to the increase of SCFAs production bacteria by improving inflammation and maintaining gut barrier integrity. In addition, SYN showed a clear synergistic effect in the SCFAs results (Table 2), which appears to have helped improve the cognitive function, as well as the intestine. In addition, SYN showed a clear synergistic effect in the SCFAs, and in particular, it seems to help in improving the cognitive function by increasing the production of acetic acid and butyric acid, as well as the effect on the gut.

Gut damage caused by intestinal inflammation and dysbiosis is associated with cognitive function development and damage [6]. Previous studies have shown an increased incidence of cognitive impairment, such as short-term and long-term memory function disorder, verbal ability disorder, and depression in IBD patients [8,25]. Furthermore, it had been reported that gut-derived immune-active cytokines caused neuronal loss and amyloid plaque accumulation in the hippocampus of DSS-induced colitis mice [54]. Indeed, inflammation from the gut can interact with the brain, increase oxidative stress, and cause neuronal damage [44]. These findings suggest that colonic inflammation can cause cognitive dysfunction. In this study, the intake of WCF and *L. reuteri* prevented cognitive impairment in behavior tests (Figure 5). In particular, in the passive avoidance test used as a behavioral test for short-term memory, the SYN group showed improved memory ability compared to the PRE and PRO groups. Consequently, these results suggest that PRE not only has individual activity, but also a synergistic effect with PRO. Meanwhile, *Lactobacillus* sp. and cornuside in *C. fructus* ameliorated the long-term memory in the Morris water maze test, learning memory in the passive avoidance test, and working memory in the Y-maze test, in scopolamine-induced cognitive deficit mice [55,56]. In addition, *L. reuteri* also improved cognitive disorders related to mental health in anxiety-like behavior in the elevated plus maze task and depressive-like behavior in the forced swimming test and tail suspension test in immobilization stress-induced colitis mice [57]. Similar to those studies, PRE and PRO showed improvement in working memory and short-term memory function disorders, and, in particular, the mixture of PRE and PRO had a synergistic effect in cognitive function.

IBD patients not only have intestinal inflammation, but also dysbiosis [26]. Variation of the gut microbiome changes microbial metabolites, and this leads to reactive oxygen species (ROS) production from IECs and neutrophils [6]. The increase in ROS and imbalance of the antioxidant system due to intestinal damage can reach the brain through systemic circulation and affect the oxidation state of the CNS directly or indirectly [58]. The excessively generated pro-inflammatory and oxidative mediators induced by cell damage induce oxidative stress and DNA injury, and promote a chain reaction of membrane lipid peroxidation [59,60]. Brain membranes are vulnerable to oxidative stress because they are rich in polyunsaturated fatty acids and peroxidable fatty acids [61]. In addition, brain tissue, which consumes a proportionately higher amount of oxygen, can be damaged by excessive oxidative stress because it has a less effective antioxidant system than other organs [60]. Increased oxidative stress in the brain induces lipid peroxidation product accumulation and an imbalance of enzymatic antioxidant activity, such as SOD, catalase, and glutathione peroxidase in the host [62]. Various studies have demonstrated that lipid peroxidation and oxidative stress are important factors in early functional events of neurodegenerative disease, such as AD, Huntington’s, and Parkinson’s diseases (PD) [63,64]. Also, lipid peroxidation is known to cause necrosis and neuronal cell death by directly damaging cell membranes and generating secondary products that contain neurotoxic activity [65]. In this study, the PRE, PRO, and SYN groups reduced brain oxidative stress (Figure 6). In particular, the SOD level was higher when the mixture was administered than when PRE and PRO were administered alone. It has been reported that morroniside, the main component of *C. fructus*, decreased the level of oxidative stress by increasing the SOD activity and glutathione content in cerebral ischemic damage in rat [66]. Moreover, it is known that probiotics, such as *Lactobacillus*, not only have their own antioxidant enzymes, but also can produce antioxidant metabolites, such as SCFAs [67]. In the current study, intake of PRE and PRO increased butyric acid content in mice fecal matter (Table 2), and it has been reported that butyric acid can defend from oxidative stress by modulating catalase, SOD, and glutathione peroxidase through the Keap1/Nrf2/HO-1 antioxidant signaling pathway [68]. In addition, treatment of butyric acid has been shown to reduce ROS levels and increase glutathione (GSH) content in the colonic mucosa [69]. Thus, it has been identified that PRE and PRO have a synergetic effect on antioxidant activity from oxidative stress in brain tissue induced by DSS.

Numerous neurons that make up the brain communicate with other neurons through neurotransmitters to control nerve functions [70,71]. The neurotransmitter imbalances affect various functions of the brain, such as cognition, behavior, and mood. In addition, they can lead to neurodegenerative disorders, such as AD and PD. Neurotransmitters, such as ACh, dopamine, serotonin, and GABA, are formed in the gut microbiota, which may affect the concentration of ACh precursors in the brain [72,73]. Therefore, it is suggested that changes of the gut microbiome can affect the synthesis and metabolism of neurotransmitters and their precursors, which may contribute to cognitive decline in the brain. ACh, a neurotransmitter, is essential for brain and nervous system function, and has been reported to play a role in spatial memory learning and the formation of transient short-term memory in rodents [71]. ACh is synthesized from choline and acetyl-CoA by ChAT in the cytoplasm, and hydrolyzed by AChE present in the synaptic cleft and recycled to the presynaptic nerve endings [74]. Since the neurotransmitter acetylcholine is decreased in the brain of AD patients, it is important to increase the amount of ACh by reversibly inhibiting AChE [75]. Moreover, cholinergic dysfunction in AD is associated with the development of two major histopathological features, including Aβ plaques and tau tangle [76]. Accumulation of amyloid plaques and hyperphosphorylated tau protein are the main pathological symptoms of AD, resulting in brain neuronal damage and apoptosis by disrupting cellular calcium homeostasis [75]. In this study, the PRE, PRO, and SYN groups improved the cholinergic system and AD pathological symptoms (p-tau and Aβ), and these results showed that SYN showed more improvement effects through synergistic activity (Figure 7 and Figure 8). In particular, SYN was more effect in AChE activity and ACh content. In a previous in vitro study, 7-O-galloyl-D-sedoheptulose, loganin, and morroniside isolated from *C. fructus* inhibited AChE and β-site amyloid precursor protein cleaving enzyme 1 (BACE1) [14]. In addition, cornuside isolated from *C. fructus* ameliorated cognitive impairment by increasing ACh level and ChAT activity and decreasing AChE activity in scopolamine-induced AD mice [55]. It has been demonstrated that *L. plantarum* MTCC 1325 improves the ACh content in the hippocampus and cerebral cortex in D-galactose-induced AD rats [77]. Moreover, probiotic supplements with *Lactobacillus* and *Bifidobacterium* protected damaged neurons, decreased Aβ plaques, and increased ACh content in the hippocampus in Aβ-induced AD rats [78]. Therefore, the cognitive function improvement effect of PRE, PRO, and SYN is considered to be derived from improvement of the cholinergic system and a decrease in the expression of Aβ and p-tau protein.

Neuronal cell death caused by apoptosis is a common characteristic in AD patients [79]. Various studies have already established that the gut microbiome plays an important role in the regulation of brain function [21,50]. IBD patients have high levels of circulating inflammatory cytokines, which can cross the BBB and affect the apoptosis pathway by increasing nitric oxide (NO) and oxygen radicals [6]. Moreover, increased intestinal permeability due to gut damage raises the levels of toxins, such as NO, ROS, and LPS, absorbed into the body [36,51]. Therefore, gut microbiome imbalance can contribute to neuronal damage in the brain via the gut-brain axis by generating harmful microbial metabolites [74]. Apoptosis is mediated through regulation of the anti-apoptotic Bcl2 protein, pro-apoptotic-Bax protein, and increased cytochrome c released into the cytoplasm, which in turn activates caspase [80]. Neuronal cell death from the apoptosis pathway contributes to neuronal damage in AD, and thus the Bcl2 family and caspase inhibition may delay Alzheimer’s pathology. Therefore, the modulating effects of PRE and PRO on the apoptosis pathway (Figure 9) may be linked to neuroprotective effects. It had been reported that cornuside, the main compound of *C. fructus*, attenuates apoptosis by regulating caspase-3 in rat cortical neurons [81]. Morroniside has a protective effect on H_2_O_2_-induced SH-SY5Y (human neuroblastoma cell line) cytotoxicity [82]. Also, morroniside decreased caspase-3 expression in ischemic cortex tissues [66]. *Lactobacillus* is known to be an antioxidant probiotic, suggesting that it may reduce cell damage caused by oxidative stress, ultimately preventing apoptosis [67]. Moreover, *L. reuteri* is an anti-inflammatory bacteria that can reduce the expression of inflammatory cytokines produced by the gut and thereby regulate neuronal cell death [18,39,40]. Therefore, the memory improvement effect of *L. reuteri* shown in this study is thought to be due to its immune-modulatory properties that can regulate inflammatory cytokines that cause neurotoxicity [83]. In summary, it is considered that the cognitive dysfunction improvement effect of PRE and PRO was due to protection from oxidative stress and improvement of cholinergic disorders caused by reduced expression of apoptotic protein and Aβ and p-tau protein.

## 4. Materials and Methods

### 4.1. Chemicals

De Man-Rogosa-Sharp (MRS) broth was purchased from MBcell (Seoul, Republic of Korea). Hexadecyltrimethylammonium bromide (HTAB), and DSS (molecular weight of 40 kDa) were purchased from Alfa Aesar (Haverhill, MA, USA). Primary antibodies for TLR 4 (sc-52962), p-IκB-α (sc-8404), TNF-α (sc-33639), caspase 1 (sc-392736), IL-1β (sc-515598), COX-2 (sc-376861), AChE (sc-373901), p-Tau (sc-12952), Aβ (sc-374527), Bcl2 (sc-509), Bax (sc-7480), and β-actin (sc-69879) were purchased from Santa Cruz Biotechnology (Dallas, CA, USA). Primary antibodies for ChAT (20747-1-AP) and iNOS (18985-1-AP) were purchased from Proteintech (Chicago, IL, USA). Primary antibodies for caspase 7 (CUB-PA05689A0Rb) was purchased from Cusabio Technology (Wuhan, China). p-NF-κB (#3033), secondary antibodies for anti-mouse IgG (#7076), and anti-rabbit IgG (#7074) were purchased from Cell Signaling Technology (Danvers, MA, USA). SOD determination kit was purchased from Dojindo Molecular Technologies (Rockville, MD, USA). FITC-dextran (4 kDa), 2-thiobarbituric acid (TBA), 2-ethylbutyric acid, standard of acetic acid, propionic acid, butyric acid, and all other chemicals used were purchased from Sigma-Aldrich Chemical Co. (St. Louis, MO, USA).

### 4.2. Sample Preparation

*C. fructus* grown in Gurye (Republic of Korea) on 22 July 2020, was dried at 50 °C. Powdered *C. fructus* was extracted in 50-fold water for 2 h at 40 °C. The extract was filtered and concentrated using a rotary evaporator (N-N series, Eyela Co., Tokyo, Japan). The WCF was used as prebiotics and stored at −80 °C until use.

*L. reuteri* KCTC 3594 was purchased from the Korean Collection for Type Cultures (KCTC, Jeongeup, Republic of Korea). To administrate *L. reuteri* to the mice, *L. reuteri* were cultured in MRS broth at 37 °C for 24 h incubation. *L. reuteri* centrifuged at 13,000× *g* for 10 min were washed twice using phosphate-buffered saline (PBS) and resuspended in PBS. To determine the number of *L. reuteri*, the logarithmic growth phase of the strain was measured absorbance at 660 nm (Epoch 2, BioTek Instruments Inc., Winooski, VT, USA). The concentrated cultures were diluted with PBS, and a final concentration was adjusted to be 1 × 10^9^ CFU/mL.

### 4.3. Animals and Experimental Design

C57BL/6 mice (6 weeks old, male) were purchased from Samtako (Osan, Republic of Korea), and the mice were randomly assigned to 3 per cage. After adaptation for 1 week, the mice were randomly divided into 5 groups (n = 9) as normal control (CON) group, DSS-induced colitis (DSS) group, WCF supplementation (300 mg/kg body weight) with DSS-induced colitis (PRE) group, *L. reuteri* supplementation (1 × 10^9^ CFU/mL) with DSS-induced colitis (PRO) group, and synbiotic supplementation containing WCF (300 mg/kg body weight) and *L. reuteri* (1 × 10^9^ CFU/mL) with DSS-induced colitis (SYN). The dose of concentration of PRE and PRO were determined from previous studies [84,85]. The mice were treated with each diet by oral gavage for 3 weeks, and then they were provided 2% (*w*/*v*) DSS with autoclaved drinking water for 6 days without CON group. An average 16.67% (DSS group, 33.33%; PRE group, 11.11%; PRO group, 11.11%; SYN group, 11.11%) of mice died during the DSS administration and behavioral experiments period. All animal experimental procedures were approved by the Institutional Animal Care and Use Committee (IACUC) of Gyeongsang National University guidelines (Certificate No. GNU-210216-M0020) on 16 February 2021.

### 4.4. Analysis of Serum Albumin

Blood samples were taken from the abdominal vein of mice after fasting for 12 h. The collected blood samples were centrifuged at 10,000× *g* for 10 min at 4 °C, and then albumin levels were detected using a biochemical analyzer (Fuji dri-chem 4000i; Fuji Film Co., Tokyo, Japan).

### 4.5. Intestinal Permeability Assay

Intestinal permeability was estimated using the FITC-dextran permeability assay. Mice were fasted for 6 h and orally treated with 60 mg/mL FITC-dextran solution (4 kDa) at a dose of 0.4 mg/g body weight. The mice were sacrificed with CO_2_ after 4 h. Blood samples were collected from the postcaval vein and immediately centrifuged 10,000× *g* for 10 min at 4 °C. The supernatant fluorescence was measured on a fluorometer at excitation wave 485 nm and emission wave 535 nm (Infinite 200, Tecan Co., Männedorf, Switzerland). FITC-dextran conctent in serum was measured using a standard curve created serially diluted in PBS.

### 4.6. Western Blot

The protein concentration of supernatant was quantified by Bradford reagent (Bio-Rad, CA, USA) methds [86]. Brain and colon tissues were homogenized with cold lysis buffer (ProtinEx Animal cell/tissue, GeneAll Biotechnology, Seoul, Republic of Korea) containing 1% protease inhibitor cocktails (Thermo Fisher Scientific, Waltham, MA, USA). The homogenates were centrifuged at 13,000× *g* at 4 °C for 10 min. The samples were separated on a 8% to 15% SDS polyacrylamide gel and transferred onto a polyvinylidene fluoride (PVDF) membrane (Millipore, Billerica, MA, USA). After, the membrane was blocked by 5% skim milk and incubated in tris-buffered saline containing 0.1% Tween 20 (TBST), including diluted primary antibody overnight at 4 °C. Then, the membrane was washed twice with TBST and incubated with secondary antibody at room temperature for 1 h. The intensity of bands obtained in the enhanced chemiluminescence (ECL)-exposed membrane was measured with an iBright CL1000 imager (Thermo Fisher Scientific). Band intensities were calculated by using Image J software (National Institutes of Health, Bethesda, MD, USA).

### 4.7. MPO Activity in Colon Tissues

Colon tissue was homogenized with 0.5% HTAB in 50 mM phosphate buffer (pH 6.0) and centrifuged at 15,000× *g* for 15 min at 4 °C. The supernatants were mixed with 50 mM potassium phosphate buffer (pH 6.0) containing o-dianisidine dihydrochloride (TCI, Tokyo, Japan) and 0.0005% hydrogen peroxide. The absorbance was detected at 450 nm with a microplate reader (Epoch 2, BioTek, Winooski, VT, USA). MPO activity was detected in units (U) of MPO/mg tissue, considering that 1 unit was defined as the amount required to disassemble 1 μmol of peroxide/min [87].

### 4.8. Behavioral Test

#### 4.8.1. Y-Maze Test

The Y-maze was constructed in a three-arms with equal angles between all white polyvinyl plastic arms. Each arm of the maze randomly consisted of A, B, and C. Mice were initially placed within a designated one of the arms, and movements of mice were recorded using a smart 3.0 video tracking system (Panlab, Barcelona, Spain) for 8 min [88].

#### 4.8.2. Passive Avoidance Test

The passive avoidance test was performed in an acryl chamber with a lighted and darkened chamber, separated by a central door. On the first day, mice were initially located in the lighted chamber for 2 min, and then mice received an electric shock (0.5 mA, 3 s) when they entered the dark chamber. On the second day, the latency time to enter the dark chamber was recorded for a maximum of 300 s [88].

### 4.9. Antioxidant Pparameters in Brain Tissue

#### 4.9.1. MDA Content

The MDA content was examined by mearing TBA reactive substance formation [89]. The brain tissue with PBS was centrifuged at 2450× *g* for 10 min. The supernatant was reacted with 1% (*v*/*v*) phosphoric acid and 0.67% (*v*/*v*) TBA at 95 °C for 1 h. The reactant was centrifuged at 600× *g* for 10 min to obtain a supernatant, and the absorbance of supernatants was detected using a microplate reader (Epoch 2, Winooski, VT, USA) at 532 nm.

#### 4.9.2. SOD Level

To measure the SOD content, the homogenized tissue in PBS was centrifuged at 400× *g* for 10 min. Cell extraction buffer (10% SOD buffer, 0.4% Triton X-100 and 200 μM phenylmethane sulfonylfluoride) was added in the obtained pellet. The extract was placed on ice for 30 min and centrifuged at 10,000× *g* for 10 min. Then, the obtained supernatant was used for the experiment. SOD content was determined using a kit following the manufacturer’s instructions.

### 4.10. Cholinergic System Activity in Brain Tissue

#### 4.10.1. ACh Level

ACh content was estimated by Hestrin’s method with slight modifications [90]. The brain tissue was homogenized in PBS and centrifuged at 12,000× *g* for 10 min. Then, the supernatant was gently mixed with 3.5 N sodium hydroxide and 2 M hydroxylamine in HCl at room temperature for 1 min. Then, the mixture was reacted with 0.5 N HCl and 0.37 M FeCl_3_. The absorbance was measured at 540 nm using a microplate reader (Epoch 2, Winooski, VT, USA).

#### 4.10.2. AChE Activity

AChE activity was measure by Ellman’s method with slight modifications [91]. The brain tissue was homogenized in PBS and centrifuged at 12,000× *g* for 10 min. Then the supernatant, as above, was mixed with 50 mM sodium phosphate buffer (pH 8.0) at 37 °C for 15 min. After reaction, Ellman’s reaction mixture was added, and the absorbance was detected at 405 nm using microplate reader (Epoch 2, Winooski, VT, USA).

### 4.11. Determination of SCFAs

The fecal samples dissolved in 5 mM NaOH were homogenized and centrifuged at 12,000× *g* for 10 min. Obtained supernatant was reacted with propanol/pyridine mixture solvent (*v*/*v* = 3:2) and propyl chloroformate with internal standard (2-ethylbutyric acid). Mixture was vortexed and derivatized by sonication for 1 min. The reaction mixture was mixed with hexane and centrifuged at 15,000× *g* for 10 min. The extracted hexane layer was used to evaluate the SCFAs level using gas chromatography Shimadzu GC-2010 Plus instrument (Shimadzu, Tokyo, Japan) and DB-5MS column (thickness, 0.25 μm; length, 30 m; diameter, 0.25 mm, Aglient, Santa Clara, CA, USA). The conditions were as follows: split ratio, 50:1; injection temperature, 260 °C; column oven temp, 40 °C; flow rate, 1.0 mL/min. He was used as the carrier gas, and the total program time was set to 16.50 min.

### 4.12. 16S rRNA Sequencing

The fecal samples were collected from mice and stored at −70 °C until use. 16S rRNA sequencing of fecal sample was performed using Illumina Miseq platform by Sanigen Co., Ltd. (Anyang, Republic of Korea). It was amplified through polymerase chain reaction (PCR) (T100 Thermal Cycler, Bio-Rad, CA, USA) using primers targeting the 16S rRNA V3-V4 region. The conditions of PCR are as follows: initial denaturation at 95 °C for 3 min, followed by 25 cycles of denaturing at 95 °C for 30 s, annealing at 55 °C for 30 s, elongation at 72 °C for 30 s, and a final extension at 72 °C for 5 min.

Paired-end MiSeq Illumina reads (2 × 300 bp) were processed using QIIME2 (version 2020.08), and the quality of the raw data was checked using FastQC (ver. 0.11.8). After quality inspection, artificial sequences, such as adapters and primers and noise, were removed. In the library prep process, the chimeric sequence intervening in the PCR process was removed to obtain high-quality sequences [92,93]. Then, the sequence data were classified down to the species level using the Sliva 16S rRNA database.

### 4.13. Identification of Bioactive Compounds

The main phenolic compound analysis of WCF was analyzed using an UPLC-Q-TOF/MS^2^. A UPLC system (Vion, Waters Corp., Milford, MA, USA) was used and analytical samples were injected into an Acquity UPLC BEH C_18_ column (100 mm × 2.1 mm × 1.7 µm) (Waters Corp., Milford, MA, USA). WCF was analyzed using distilled water containing 0.1% formic acid and acetonitrile (ACN) containing 0.1% formic acid at a flow rate of 0.6 mL/min for 12 min. The compounds separated on the column were analyzed in negative ionization mode. The data from the UPLC were analyzed using UNIFI software (Waters Corp., Milford, MA, USA).

### 4.14. Statistical Analysis

All data were presented as mean ± SD. Statistical analysis was analyzed using one-way analysis of variance (ANOVA), followed by Duncan’s multiple range test by SAS program (Ver. 9.4 SAS Institute, Cary, NC, USA). Statistical difference (*p* < 0.05) of each group was indicated by different small letters.

## 5. Conclusions

In conclusion, this study indicates that a mixture (SYN) of WCF and *L. reuteri* might improve DSS-induced colitis and cognitive impairment. These results suggest that SYN not only modulates dysbiosis and SCFAs production, but also exerts beneficial effects through intestinal immune response and intestinal barrier protection. The modulating effects of SYN in gut microbiota and gut inflammation improved cognitive behavioral disorder by improving cholinergic system disorder, cerebral antioxidant activity, and neuronal apoptosis through the gut-brain axis. This study suggests that SYN can be used as a preventive strategy for colitis and subsequent cognitive impairment by mediating the gut–brain axis balance.

## Figures and Tables

**Table 1:** Effect of PRE, PRO, and SYN on gut microbiome composition relative abundance of DSS-induced colitis mice.

	CON	DSS	PRE	PRO	SYN
Phylum					
*Proteobacteria*	0.08 ± 0.01 ^b^	9.19 ± 2.87 ^a^	0.31 ± 0.01 ^b^	1.75 ± 0.40 ^b^	0.63 ± 0.25 ^b^
*Bacteroidota*	39.68 ± 3.30 ^b^	52.45 ± 3.51 ^a^	44.01 ± 3.49 ^ab^	43.43 ± 3.44 ^ab^	49.03 ± 6.64 ^a^
*Firmicutes*	40.05 ± 9.57 ^b^	37.15 ± 7.95 ^b^	59.62 ± 5.54 ^a^	37.27 ± 0.75 ^b^	46.63 ± 0.92 ^b^
*Firmicutes/Bacteroidota* ratio	1.39 ± 0.09 ^a^	0.61 ± 0.09 ^c^	1.36 ± 0.09 ^a^	0.90 ± 0.05 ^b^	1.04 ± 0.09 ^b^
Family					
*Bacillaceae*	0.06 ± 0.00 ^b^	6.93 ± 3.08 ^a^	3.63 ± 0.58 ^ab^	5.13 ± 1.28 ^a^	3.85 ± 2.24 ^ab^
*Lachnospiraceae*	26.68 ± 4.13 ^ab^	14.62 ± 5.70 ^b^	30.86 ± 2.46 ^a^	16.89 ± 6.95 ^ab^	29.19 ± 3.32 ^ab^
*Lactobacillaceae*	5.56 ± 2.82 ^a^	0.46 ± 0.04 ^b^	1.35 ± 0.71 ^b^	6.05 ± 4.05 ^a^	1.98 ± 1.81 ^b^
Genus					
*Bacillus*	0.06 ± 0.00 ^b^	6.14 ± 2.47 ^a^	3.46 ± 0.49 ^ab^	4.74 ± 0.99 ^a^	3.19 ± 1.53 ^b^
*Prevotellaceae* UCG-001	1.01 ± 0.74 ^b^	2.31 ± 0.28 ^a^	0.81 ± 0.39 ^b^	N.D.	0.65 ± 0.09 ^b^
*Odoribacter*	0.31 ± 0.28 ^a^	0.04 ± 0.00 ^b^	0.10 ± 0.03 ^ab^	0.10 ± 0.02 ^ab^	0.11 ± 0.01 ^ab^
*Lactobacillus*	8.89 ± 4.58 ^a^	0.70 ± 0.23 ^b^	2.42 ± 1.36 ^ab^	7.41 ± 4.57 ^a^	2.77 ± 2.82 ^ab^
*Lactococcus*	0.53 ± 0.02 ^ab^	0.23 ± 0.24 ^b^	0.98 ± 0.02 ^a^	0.65 ± 0.12 ^ab^	0.87 ± 0.19 ^a^
*Lachnospiraceae* NK4A136 group	2.90 ± 0.80 ^ab^	1.43 ± 0.64 ^bc^	3.59 ± 0.75 ^a^	0.41 ± 0.03 ^c^	1.68 ± 0.76 ^abc^
*Anaerotruncus*	0.66 ± 0.16 ^a^	0.06 ± 0.04 ^b^	0.14 ± 0.00 ^b^	0.81 ± 0.27 ^a^	0.19 ± 0.10 ^b^
*Akkermansia*	0.43 ± 0.01 ^b^	0.21 ± 0.11 ^b^	0.97 ± 0.44 ^ab^	0.49 ± 0.13 ^b^	2.22 ± 0.93 ^a^
Species					
*Akkermansia muciniphila*	0.45 ± 0.04 ^d^	0.30 ± 0.16 ^e^	0.85 ± 0.40 ^b^	0.55 ± 0.18 ^c^	3.35 ± 0.19 ^a^
*Alistipes onderdonkii*	0.33 ± 0.02 ^ab^	0.02 ± 0.04 ^c^	0.34 ± 0.00 ^a^	0.19 ± 0.08 ^b^	0.28 ± 0.05 ^ab^

The results were presented as the mean ± SD (*n* = 3). Data were statistically considered at *p* < 0.05, and different lowercase letters indicate significant differences between groups. N.D.: not detected.

**Table 2:** Effect of PRE, PRO, and SYN on concentration of short-chain fatty acids (SCFAs) concentration in DSS-induced mice fecal (Unit: mM/g).

	CON	DSS	PRE	PRO	SYN
Acetic acid	182.93 ± 16.85 ^b^	81.76 ± 31.91 ^c^	216.39 ± 39.46 ^ab^	200.44 ± 42.16 ^b^	254.22 ± 39.50 ^a^
Propionic acid	18.44 ± 3.93 ^b^	8.85 ± 4.43 ^c^	28.04 ± 2.44 ^a^	25.79 ± 3.44 ^a^	30.12 ± 6.38 ^a^
Butyric acid	39.47 ± 12.68 ^a^	7.28 ± 3.13 ^d^	23.20 ± 2.31 ^c^	24.83 ± 5.42 ^bc^	38.18 ± 10.89 ^ab^

The results were presented as the mean ± SD (*n* = 5). Data were statistically considered at *p* < 0.05, and different lowercase letters indicate significant differences between groups.

**Table 3:** Identification of main compounds in the ethyl acetate fractions from *Corni fructus* (WCF) by UPLC-Q-TOF/MS^2^ system.

Compound	Retention Time (min)	Formular	[M-H] (*m*/*z*)	Production (*m*/*z*)
Quinic acid	0.81	C_7_H_12_O_6_	191	85, 93, 109, 127, 191
Morroniside	4.49	C_17_H_26_O_11_	451	101, 123, 141, 155, 243, 451
Loganin	4.69	C_17_H_26_O_10_	435	101, 127, 209, 227, 435
Cornuside	5.30	C_24_H_30_O_14_	541	125, 169, 347, 541

## Data Availability

The data presented in this study are available on request from the corresponding author applicable.

## Supplementary Materials

The following supporting information can be downloaded at: https://www.mdpi.com/article/10.3390/ijms24010090/s1, Figure S1: Effects of PRE, PRO, and SYN on gut microbiome composition of DSS-induced colitis mice. Taxonomic breadth of bacteria visualized using Krona plot of CON group (**a**), DSS group (**b**), PRE group (**c**), PRO group (**d**), and SYN group (**e**).
