# Supplementary material for: Anti-Amnesic Effect of Synbiotic Supplementation Containing Corni fructus and Limosilactobacillus reuteri in DSS-Induced Colitis Mice
Source: Int J Mol Sci. 2022 Dec 21;24(1):90. doi: 10.3390/ijms24010090 (PMC9820465; doi:10.3390/ijms24010090)
Supplement: Supplementary file 1 [file ijms-24-00090-s001.zip › ijms-2069618-supplementary.pdf]

a

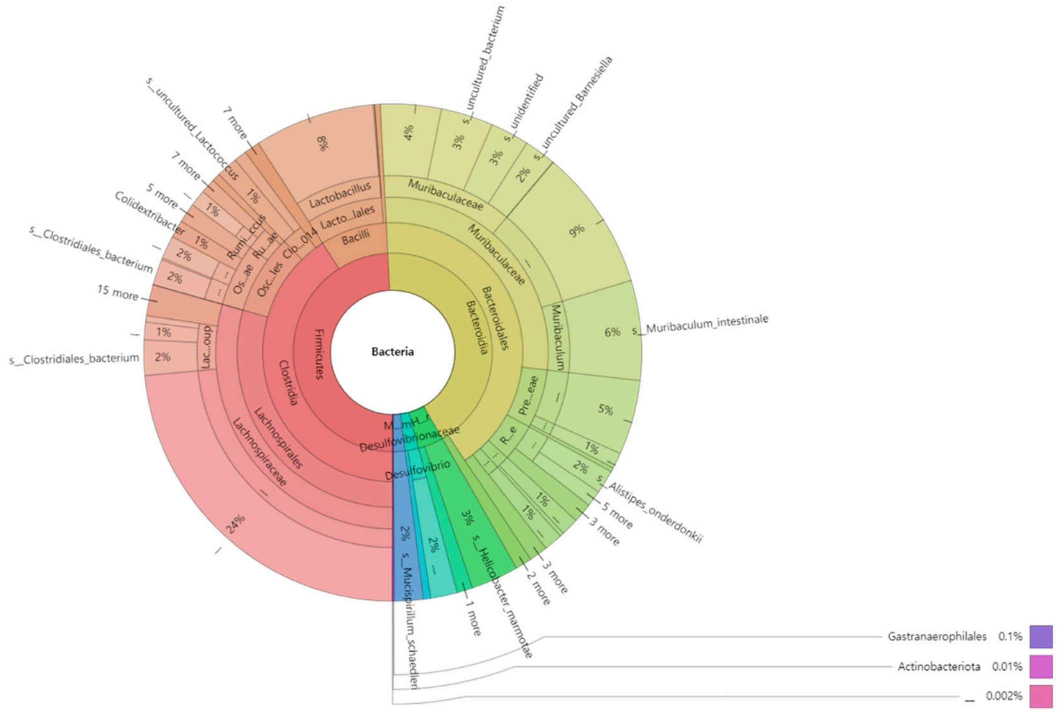

Bacteria
Firmicutes
Clostridia
Lachnospirales
Lachnospiraceae
24%
Bacilli
Lacto...lales
Lactobacillus
8%
Bacteroidia
Bacteroidales
Muribaculaceae
Muribaculum
s__uncultured_bacterium
s__unidentified
s__uncultured_Barnesiella
s__Muribaculum_intestinale
s__Alistipes_onderdonkii
s__Helicobacter_marmotae
s__Mucispirillum_schaedleri
s__Clostridiales_bacterium
s__uncultured_Lactococcus
Colidextribacter
Desulfovibrionaceae
Desulfovibrio
15 more
7 more
5 more
3 more
2 more
1 more
Gastranaerophilales 0.1%
Actinobacteriota 0.01%
__ 0.002%

b

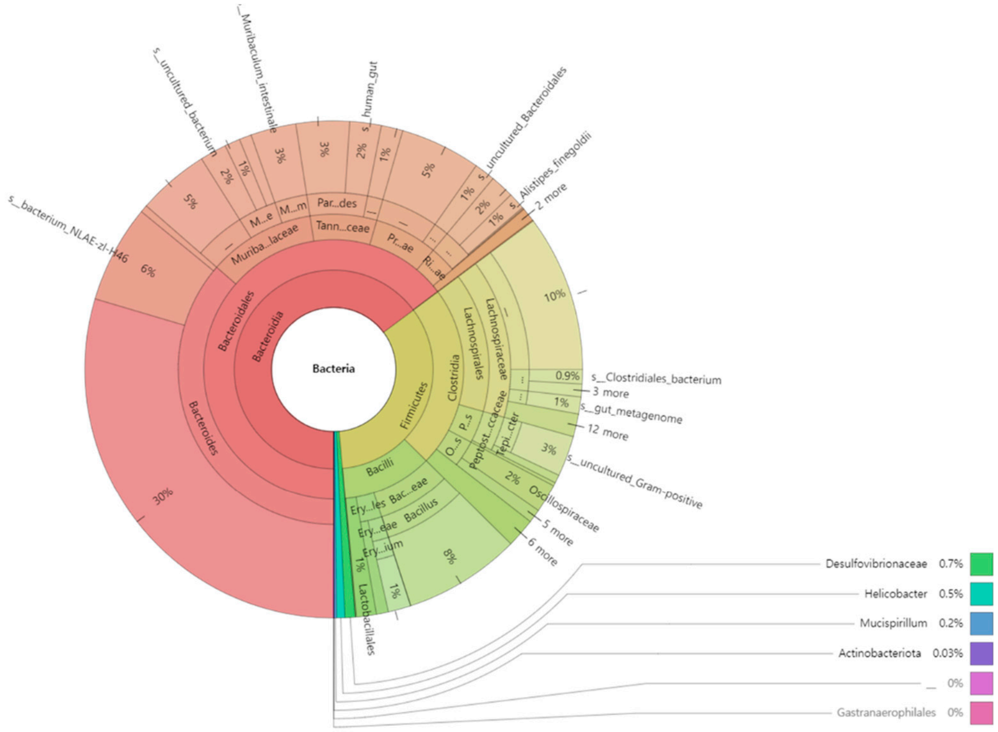

Bacteria
Bacteroidia
Bacteroidales
Bacteroides
30%
Muriba...laceae
Tann...ceae
Par...des
s__bacterium_NLAE-zl-H46
s__uncultured_bacterium
s__Muribaculum_intestinale
s__human_gut
s__uncultured_Bacteroidales
s__Alistipes_finegoldii
2 more
Firmicutes
Clostridia
Lachnospirales
Lachnospiraceae
Peptost...ccaceae
Oscillospiraceae
Bacilli
Bacillus
Lactobacillales
s__Clostridiales_bacterium
s__gut_metagenome
s__uncultured_Gram-positive
12 more
6 more
5 more
3 more
10%
8%
Desulfovibrionaceae 0.7%
Helicobacter 0.5%
Mucispirillum 0.2%
Actinobacteriota 0.03%
__ 0%
Gastranaerophilales 0%

c

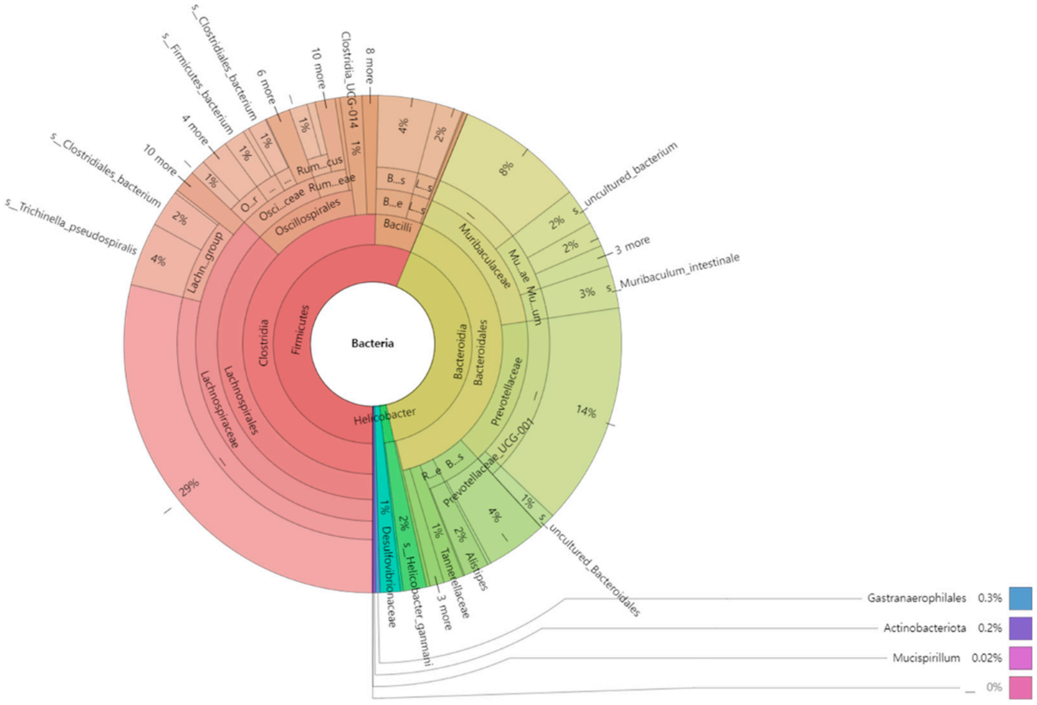

Bacteria
Firmicutes
Clostridia
Lachnospirales
Lachnospiraceae
Bacteroidia
Bacteroidales
Muribaculaceae
Prevotellaceae
Prevotellaceae_UCG-001
Helicobacter
Desulfovibrionaceae
Tannerellaceae
Alistipes
Oscillospirales
Bacilli
s__Trichinella_pseudospiralis
s__Clostridiales_bacterium
s__Firmicutes_bacterium
Clostridia_UCG-014
s__uncultured_bacterium
s__Muribaculum_intestinale
s__uncultured_Bacteroidales
s__Helicobacter_ganmani
29%
14%
8%
Gastranaerophilales 0.3%
Actinobacteriota 0.2%
Mucispirillum 0.02%
0%

d

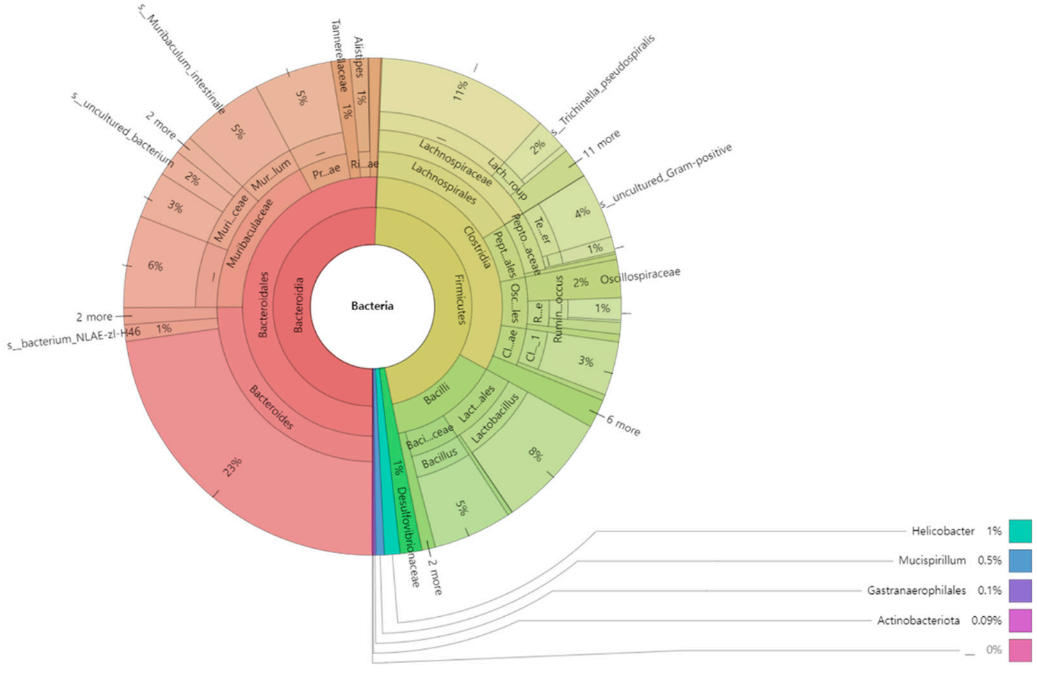

Bacteria
Bacteroidia
Bacteroidales
Bacteroides
Muribaculaceae
Firmicutes
Clostridia
Lachnospirales
Lachnospiraceae
Oscillospiraceae
Bacilli
Lactobacillus
Bacillus
Desulfovibrionaceae
Tannerellaceae
Alistipes
s__Muribaculum_intestinale
s__uncultured_bacterium
s__bacterium_NLAE-zl-H46
s__Trichinella_pseudospiralis
s__uncultured_Gram-positive
23%
11%
8%
Helicobacter 1%
Mucispirillum 0.5%
Gastranaerophilales 0.1%
Actinobacteriota 0.09%
0%

e

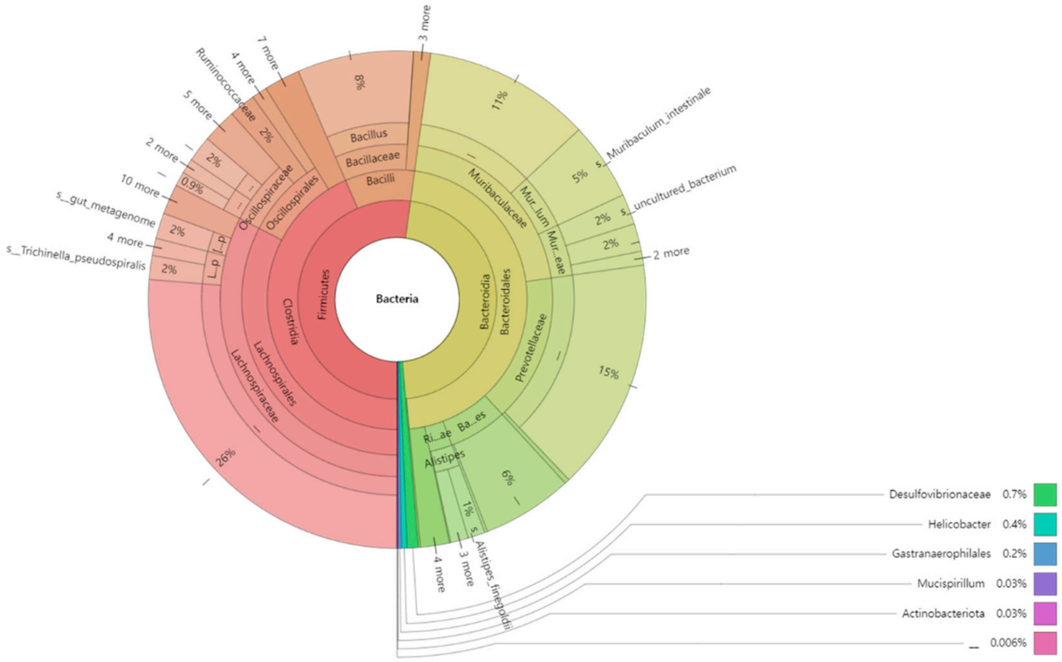

Supplementary Figure S1. Effects of PRE, PRO, and SYN on gut microbiome composition of DSS-induced colitis mice. Taxonomic breadth of bacteria visualized using Krona plot of CON group(**a**), DSS group (**b**), PRE group (**c**), PRO group (**d**), and SYN group (**e**). The results were shown with mean ± SD ($n = 3$). Data were statistically considered at $p < 0.05$, and different small letters represented a statistical difference.
